# Systemic Sarcoidosis Mimicking Metastatic Melanoma: Unveiling the Masquerade

**DOI:** 10.7759/cureus.96912

**Published:** 2025-11-15

**Authors:** Shu Ying Chee, Daire Goodman, Aisling Nolan, Jack Kelly

**Affiliations:** 1 Department of Plastic and Reconstructive Surgery, University Hospital Galway, Galway, IRL; 2 Department of Pathology, University Hospital Galway, Galway, IRL

**Keywords:** histopathology, ireland, melanoma, multidisciplinary teams, systemic sarcoidosis

## Abstract

The coexistence of melanoma and sarcoidosis presents significant diagnostic challenges, particularly when imaging studies reveal suspicious lesions that may represent either metastatic disease or granulomatous inflammation. This complexity is compounded by the overlapping clinical and radiological features of both conditions.

We present the case of a woman in her 80s with a history of pulmonary sarcoidosis who developed a thick melanoma (5.1 mm Breslow depth) and subsequently underwent wide local excision with a negative sentinel lymph node biopsy. Surveillance imaging revealed an FDG-avid lytic lesion in the fibular head, raising suspicion for metastatic disease. Histopathological examination of the fibular lesion confirmed sarcoidosis rather than melanoma metastasis. The patient continues under clinical surveillance without evidence of melanoma progression.

This case highlights the diagnostic complexity when melanoma and sarcoidosis coexist. Multidisciplinary team discussion and histopathological confirmation remain essential for accurate diagnosis and appropriate management decisions. Given Ireland's high prevalence of both conditions, clinicians must maintain awareness of this potential diagnostic pitfall.

## Introduction

The co-occurrence of melanoma and systemic inflammatory conditions presents significant diagnostic challenges, particularly when unexpected findings arise during radiological assessments. Melanoma, a malignant tumor resulting from the transformation of melanocytes, primarily occurs in the skin but can also develop in the gastrointestinal tract and brain. Its propensity for metastasis to the lungs, liver, bones, and brain is well-documented. Conversely, sarcoidosis is a multisystem disorder of unknown etiology, characterized by non-caseating granulomas that most commonly affect the lungs, eyes, and skin, but can also involve the liver, heart, bones, and joints [1]. Skeletal involvement in sarcoidosis is relatively rare, with reported prevalence rates ranging from 3% to 13% [2]. Notably, only 50% of individuals with osseous sarcoidosis present with symptoms, suggesting that its prevalence may be underestimated. Symptoms range from arthralgia to widespread destructive bone lesions [2]. Moreover, osseous involvement typically occurs later in the disease course, and patients with osseous sarcoidosis often demonstrate involvement of three or more organ systems [3]. Ireland has the second-highest global prevalence of sarcoidosis, particularly affecting individuals aged 25 to 40 years. Studies indicate that patients with sarcoidosis have an increased risk of malignancy, especially concerning skin cancers, hematological malignancies, and leukemia [4,5]. The coexistence of systemic sarcoidosis with malignant melanoma complicates diagnosis and treatment, often leading to misinterpretation due to overlapping clinical and radiological characteristics.

## Case presentation

A woman in her 80s presented in February 2021 with a pigmented skin lesion on her left foot, which was subsequently biopsied, confirming melanoma with a Breslow thickness of 5.1 mm (pt4aN0M0). In March 2021, she underwent wide local excision and skin graft reconstruction, and her sentinel lymph node biopsy yielded negative results. Her medical history included a diagnosis of pulmonary sarcoidosis 27 years earlier, detected incidentally during a routine chest X-ray. At the time of diagnosis, she was asymptomatic and remained so, receiving no treatment for the subsequent 22 years. Given her significant comorbidities, her case was reviewed in a multidisciplinary team (MDT) meeting, leading to the recommendation for radiological surveillance, including a PET-CT scan and MRI of the brain, alongside clinical follow-up, rather than the initiation of immunotherapy.

**Figure 1:**
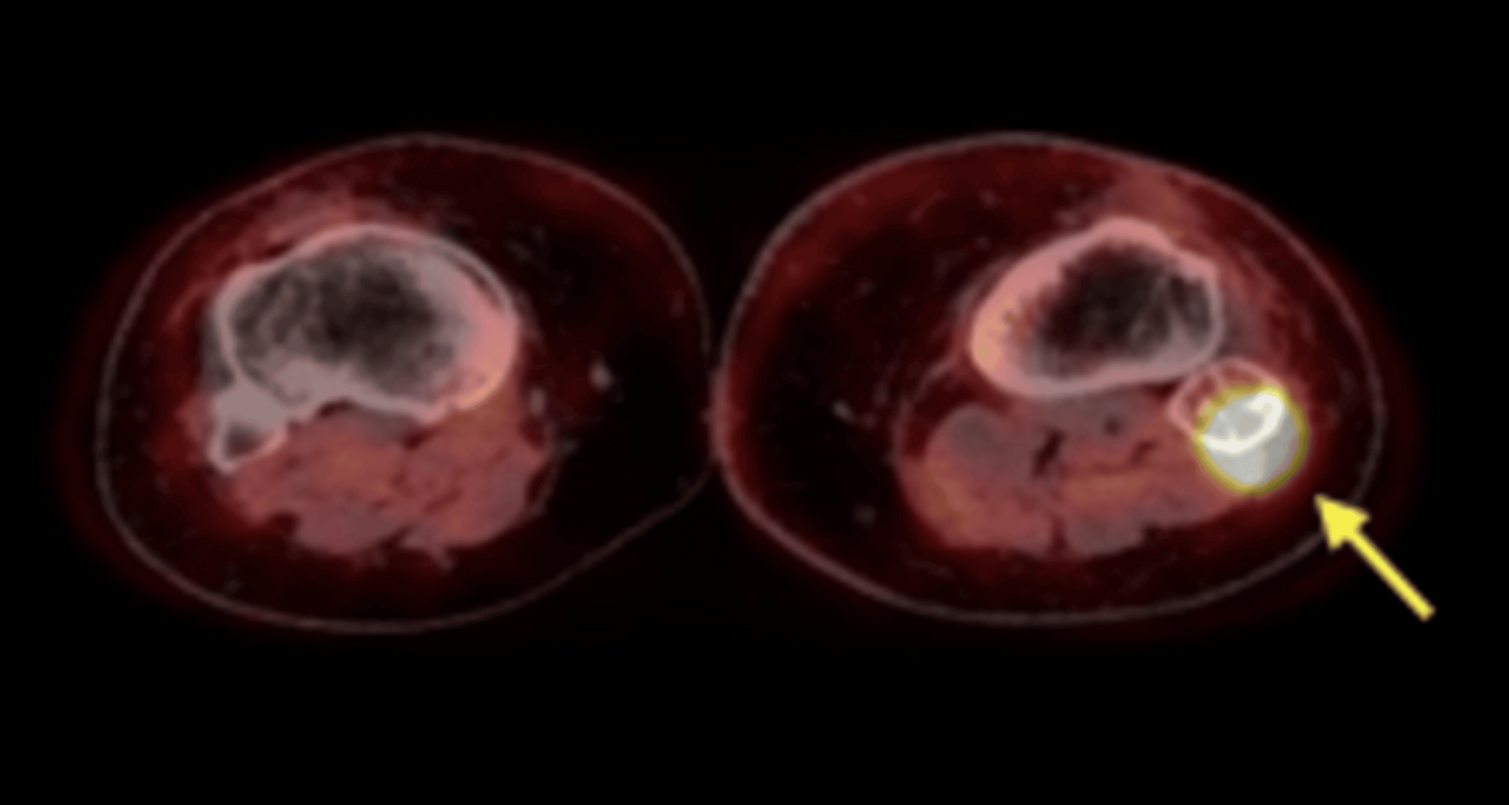
PET-CT scan (axial view): FDG-avid lytic lesion in the head of the left fibula (arrow)

**Figure 2:**
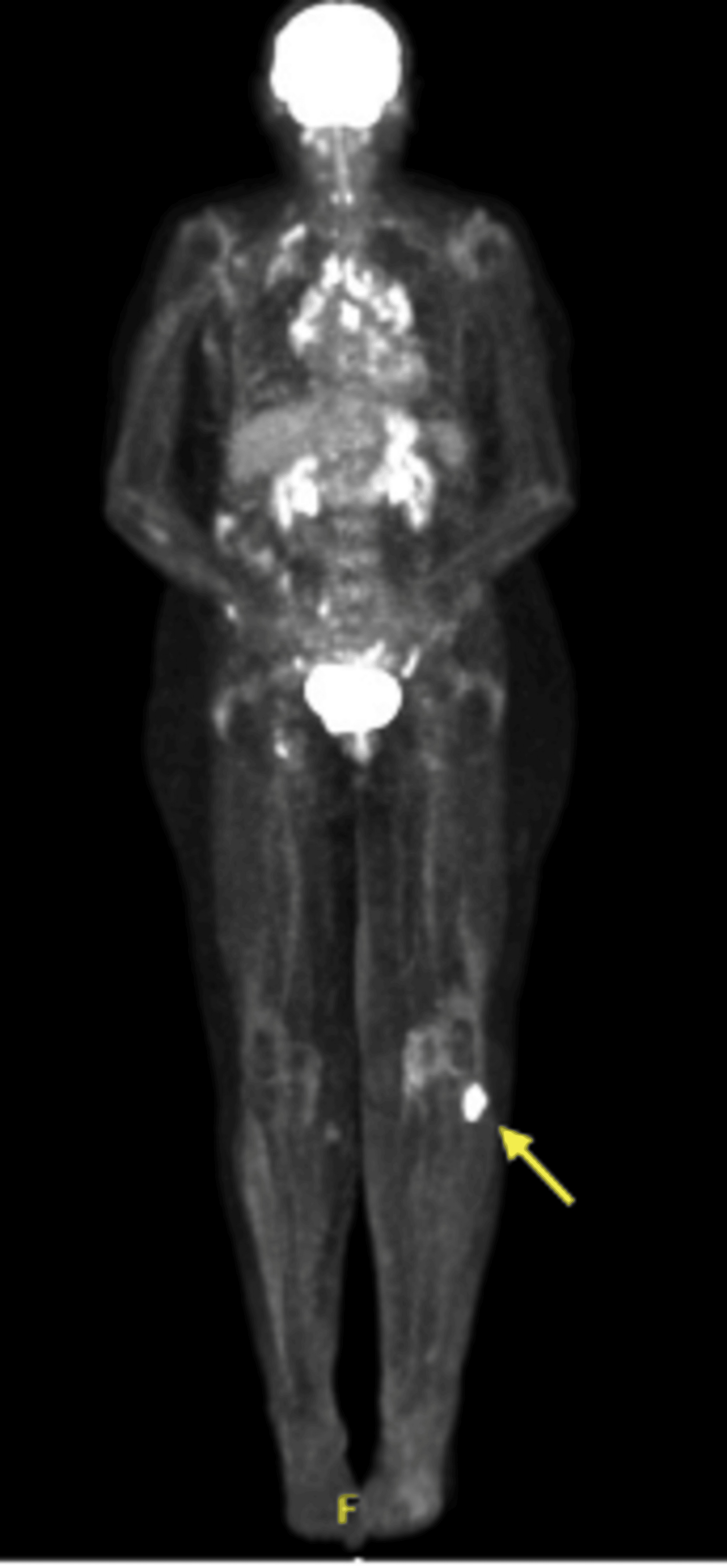
PET-CT scan (coronal view): FDG-avid lytic lesion in the head of the left fibula (arrow)

**Figure 3:**
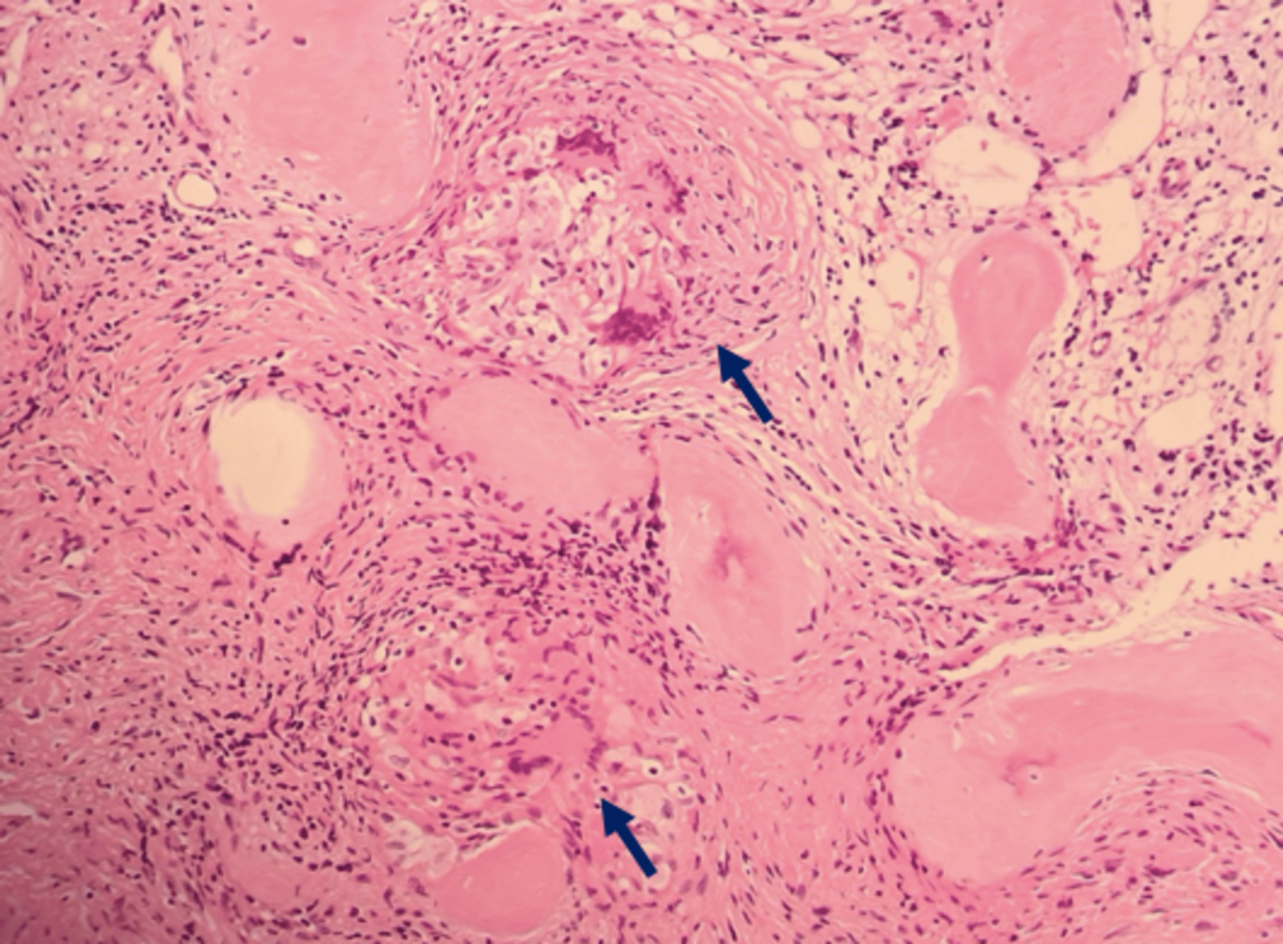
Excisional biopsy of the lesion on the left fibula (histology) showing a high-power image of non-caseating epithelioid granulomas: discrete aggregates of eosinophilic macrophages with variable giant cell formation

**Figure 4:**
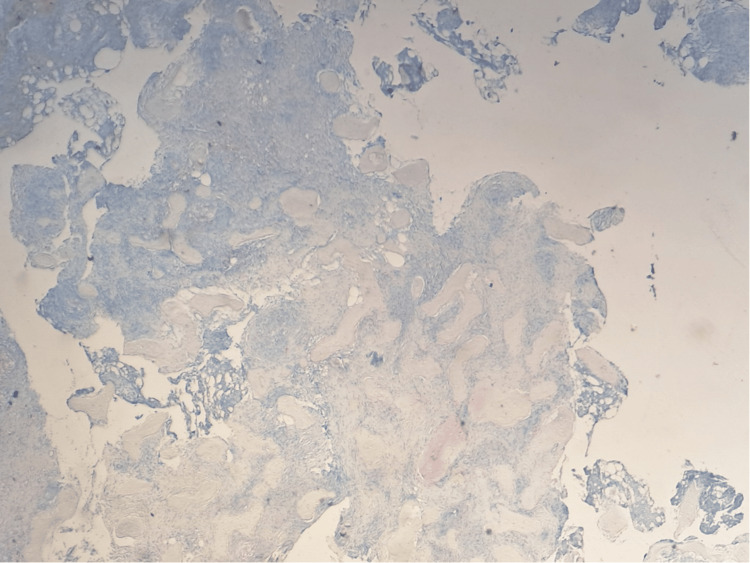
Excisional biopsy of the lesion on the left fibula (immunohistochemical staining): S100 stain negative for melanoma

**Figure 5:**
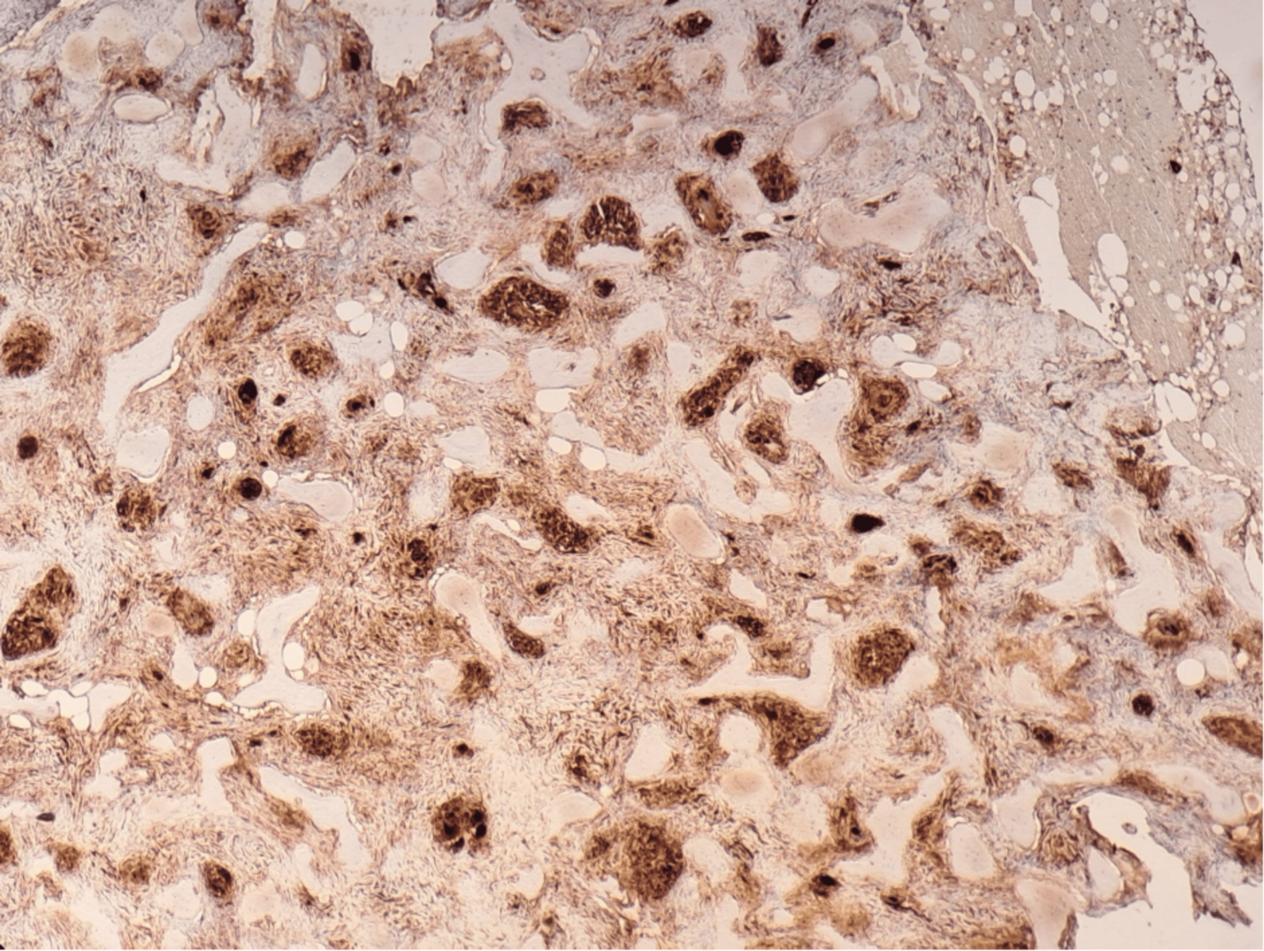
Excisional biopsy of the lesion on the left fibula (immunohistochemical staining): CD68 stain highlights the granulomas

In August 2021, a PET-CT scan revealed an FDG-avid lytic lesion in the head of the left fibula, raising suspicion of metastatic melanoma (Figures 1-2). An excisional biopsy of this lesion, performed in August 2021, confirmed the presence of sarcoidosis (Figures 3-5). The patient continues to receive clinical surveillance under our care and is doing well.

## Discussion

The coexistence of melanoma and sarcoidosis represents a well-recognized but diagnostically challenging clinical scenario that requires careful multidisciplinary evaluation [5,6]. This case exemplifies the diagnostic complexity that arises when patients with both conditions develop new lesions on surveillance imaging, particularly given the overlapping radiological features between sarcoidosis and metastatic melanoma.

The relationship between sarcoidosis and malignancy, particularly melanoma, demonstrates increasing complexity with bidirectional associations. Recent large-scale cohort studies have confirmed a significantly higher prevalence of malignancies among sarcoidosis patients compared to controls (19.5% vs 13.6%), with this association remaining robust after adjusting for potential confounders [7]. Conversely, sarcoidosis-like reactions have been increasingly recognized in melanoma patients, particularly those receiving immunotherapy or targeted therapies, with reported incidence rates of 0.58% in melanoma cohorts [8].

Radiological differentiation between osseous sarcoidosis and metastatic melanoma remains problematic. MRI features lack sufficient sensitivity and specificity to allow reliable distinction [9]. FDG-PET imaging is similarly non-specific, with granulomatous uptake mimicking metastatic disease [10,11]. These limitations highlight the indispensable role of histopathological confirmation.

Ireland’s unique epidemiological context adds complexity, as both melanoma incidence and sarcoidosis prevalence are high, necessitating greater diagnostic vigilance [12]. The Republic of Ireland demonstrates a sarcoidosis prevalence of 28.13 per 100,000, with significant spatial clustering in the Northwest (44.9 per 100,000) and Midlands regions (32.1 per 100,000) [12]. These prevalence rates are among the highest globally and are comparable to Scandinavian countries [12]. Combined with Ireland's increasing melanoma incidence, clinicians must maintain heightened awareness of the potential for coexistence of these conditions.

The MDT approach adopted in this case represents best practice for managing such complex diagnostic scenarios [13]. Recent case series emphasize the crucial role of the MDT in determining treatment courses when sarcoidosis-like reactions occur during melanoma therapy, balancing the risks of continuing or suspending cancer therapies while managing inflammatory complications [8]. The involvement of dermatologists, oncologists, radiologists, pathologists, and surgeons ensures comprehensive evaluation and appropriate clinical decision-making. The MDT's decision to pursue surveillance rather than systemic therapy in this elderly patient with significant comorbidities reflects the individualized approach necessary when managing patients with multiple complex conditions. This approach is particularly relevant given the emerging recognition of sarcoidosis-like reactions as immune-related adverse events in melanoma patients receiving checkpoint inhibitors or BRAF/MEK targeted therapies [14]. The management implications of accurate diagnosis are significant. Misinterpretation of sarcoidal lesions as metastatic disease may lead to inappropriate staging, unnecessary investigations, and potential over-treatment with systemic therapies. Conversely, failure to recognize true metastatic disease could result in delayed treatment and compromised oncological outcomes [15].

Future developments in imaging technology may improve diagnostic accuracy. Advanced MRI techniques, including diffusion-weighted whole-body MRI, show promise for better differentiation between inflammatory and malignant processes [16]. However, these techniques remain investigational and are not yet widely available for routine clinical use. The development of novel PET radiotracers specifically targeting granulomatous inflammation may also enhance diagnostic precision [17]. This case also highlights the importance of long-term surveillance strategies in patients with dual diagnoses. The appropriate surveillance interval and imaging modalities must be carefully considered, balancing the need for early detection of melanoma progression against the potential for misinterpretation of benign sarcoidal lesions. Close collaboration between specialties and clear communication of the patient's complex medical history remain essential components of successful management.

## Conclusions

Malignant melanoma and sarcoidosis are both immune-related conditions but differ fundamentally in pathogenesis. With high rates of both diseases in Ireland, this case illustrates the importance of maintaining diagnostic flexibility and leveraging MDT expertise to reach the correct management decision.
